# Enhanced Mechanical and Thermal Performance of Sustainable RPET/PA-11/Joncryl^®^ Nanocomposites Reinforced with Halloysite Nanotubes

**DOI:** 10.3390/polym17111433

**Published:** 2025-05-22

**Authors:** Zahid Iqbal Khan, Mohammed E. Ali Mohsin, Unsia Habib, Suleiman Mousa, SK Safdar Hossain, Syed Sadiq Ali, Zurina Mohamad, Norhayani Othman

**Affiliations:** 1 Faculty of Chemical and Energy Engineering, Universiti Teknologi Malaysia (UTM), Johor Bahru 81310, Johor, Malaysia; zahidiqbalkhan@utm.my (Z.I.K.);; 2 Department of Chemical Engineering, College of Engineering, King Faisal University, P.O. Box 400, Al Ahsa 31982, Saudi Arabia; 3 Department of Mechanical Engineering, College of Engineering, King Faisal University, P.O. Box 400, Al Ahsa 31982, Saudi Arabia

**Keywords:** RPET/PA-11 nanocomposites, HNT reinforcement, Joncryl^®^ compatibilization, sustainable polymers, mechanical and thermal enhancement

## Abstract

The rapid advancement of sustainable materials has driven the need for high-performance polymer nanocomposites with superior mechanical, thermal, and structural properties. In this study, a novel RPET/PA-11/Joncryl^®^ nanocomposite reinforced with halloysite nanotubes (HNTs) is developed for the first time, marking a significant breakthrough in polymer engineering. Six different proportions of HNT (0, 1, 2, 3, 4, and 5 phr) are introduced to the blend of rPET/PA-11/Joncryl^®^ through a twin-screw extruder and injection moulding machine. The incorporation of HNTs into the RPET/PA-11 matrix, coupled with Joncryl^®^ as a compatibilizer, results in a synergistic enhancement of material properties through improved interfacial adhesion, load transfer efficiency, and nanoscale reinforcement. Comprehensive characterization reveals that the optimal formulation with 2 phr HNT (NCS-H2) achieves remarkable improvements in tensile strength (56.14 MPa), flexural strength (68.34 MPa), and Young’s modulus (895 MPa), far exceeding conventional polymer blends. Impact resistance reaches 243.46 J/m, demonstrating exceptional energy absorption and fracture toughness. Thermal analysis confirms enhanced stability, with an onset degradation temperature of 370 °C, attributing the improvement to effective matrix–filler interactions and restricted chain mobility. Morphological analysis through FESEM validates uniform HNT dispersion at optimal loading, eliminating agglomeration-induced stress concentrators and reinforcing the polymer network. The pioneering integration of HNT into RPET/PA-11/Joncryl^®^ nanocomposites not only bridges a critical gap in sustainable polymers but also establishes a new benchmark for polymer nanocomposites. This work presents an eco-friendly solution for engineering applications, offering mechanical robustness, thermal stability, and recyclability. The results form the basis for next-generation high-performance materials for industrial use in automotive, aerospace, and high-strength structural applications.

## 1. Introduction

The global transition to sustainability has accelerated the development of advanced materials that combine high performance with environmental responsibility [1]. Among these, polymer nanocomposites have emerged as a promising solution to overcome the mechanical, thermal, and structural limitations of traditional polymer blends [2]. The need to recycle durable and thermally stable polymers has driven extensive research in polymer engineering [3]. Despite significant progress in this area, many polymer systems still struggle to achieve an optimal balance of properties, particularly when recycled components are integrated. One of the most common polymers used worldwide is recycled polyethylene terephthalate (RPET). It is generally known for its sustainability and mechanical properties, making it an important polymer material for reducing plastic waste. However, its inherent brittleness when recycled and limited compatibility with other polymers restrict its use in high-performance engineering materials [4,5]. Therefore, there is a dire need to enhance RPET properties by adding some polymers or additives that can improve the properties after recycling for various engineering applications. Several studies have explored the blending of recycled PET (RPET) with various polymers, including high-density polyethylene (HDPE), polypropylene (PP) [6,7], polystyrene (PS) [8], polycarbonate (PC) [9], and different types of polyamides such as PA6 [10] and PA-11 [11,12,13]. Among these, PA6 has been widely studied in combination with RPET due to its mechanical advantages [10,14]. Nonetheless, RPET/PA6 blends face notable drawbacks, such as high moisture uptake, reduced impact resistance, and a tendency for PA6 to degrade under long processing of industrial usage. These issues highlight the importance of developing improved blending methods and incorporating innovative fillers to enhance the compatibility and performance of RPET-based systems [10]. In contrast, polyamide 11 (PA-11), a bio-based polymer derived from renewable resources, has gained attention for its excellent mechanical properties, chemical stability, and flexibility [15]. When blended with RPET, PA-11 has the potential to form a sustainable matrix with enhanced thermal and mechanical performance. However, the inherent immiscibility between RPET and PA-11 frequently leads to phase separation, which can compromise the uniformity and functionality of the final material [13,16].

To overcome the compatibility issues in PET/PA6 blends, various compatibilizers and chain extenders have been explored, including Poly(isoprene-g-Maleic Anhydride) [14], Joncryl^®^ [17], and copoly(ester-amide 6) [18]. Among these, Joncryl^®^ has been widely recognized for its ability to improve the interfacial adhesion and mechanical performance of PET/PA6 systems. This is attributed to its epoxy functional groups, which can form covalent bonds with the hydroxyl and carboxyl ends of PET as well as with the amine terminals of PA6, effectively increasing molecular weight and enhancing blend homogeneity [17]. In the context of RPET/PA-11, which is a relatively new blend system, our previous research demonstrated that incorporating Joncryl^®^ as a compatibilizer and chain extender significantly improved the miscibility and overall properties of the material [16,19]. However, the resulting properties remained inadequate for many applications as it required further reinforcement to achieve the desired strength, toughness, and durability as well as thermal stability. To improve the performance of these blends, researchers have focused on, incorporating nanofillers such as sepiolite and graphene [16,20,21]. However, the incorporation of sepiolite and graphene has been found to reduce mechanical properties, with tensile strength decreasing to 37 MPa, which is very low for many applications. Additionally, nanocomposites containing sepiolite and graphene often face challenges related to filler dispersion, high production costs, and reduced efficiency. These limitations primarily arise from poor interfacial interactions between the fillers and the RPET/PA-11 matrix, as well as the tendency of fillers to agglomerate, thereby compromising the overall performance of the composite. On the other hand, of late, halloysite nanotubes (HNTs) have gained increasing attention due to their unique tubular morphology, high aspect ratio, presence of a silanol group for better interaction, and excellent dispersion properties [22]. Furthermore, HNT offers excellent thermal properties, and better interfacial interactions within polymer matrices while being cost-effective and easy to process [23]. Previous research on HNT-based nanocomposites with various polymers has proven the potential of HNT to improve the mechanical and thermal properties of various polymer systems [24,25,26]. However, there is no literature highlighting the effect of the incorporation of HNTs into an RPET/PA-11/Joncryl^®^ blend. Thus, this study pioneers a novel approach that integrates HNTs into the RPET/PA-11 polymer matrix in the presence of the Joncryl^®^ compatibilizer and optimizes its properties for high-performance applications.

This study introduces, for the first time, a novel RPET/PA-11 nanocomposite reinforced with halloysite nanotubes (HNT) and compatibilized with Joncryl^®^, representing a significant advancement in polymer nanocomposites. The key innovation lies in the unprecedented synergistic effect between Joncryl^®^ and HNT, which will enhance the structural integrity, mechanical strength, and thermal stability. Through systematic optimization and dispersion of HNTs, this research aims to achieve remarkable improvements in load transfer efficiency, interfacial adhesion, and overall material performance. The successful development of these advanced RPET/PA-11/Joncryl^®^/HNT nanocomposites will not only address the critical challenges associated with filler dispersion, miscibility, and interfacial compatibility, but will also set new benchmarks for sustainable polymer-based materials. These findings will have far-reaching implications for applications in the automotive, aerospace, and packaging industries, where high-performance, recyclable materials are in growing demand. Furthermore, the exceptional reinforcement effect of HNT and compatibilization via Joncryl^®^ provides a transformative approach to polymer recycling, paving the way for next-generation, environmentally friendly nanocomposites. The aim of this work is to establish a solid foundation for future innovations in sustainable, high-performance materials and reinforce the crucial role of nanotechnology in advancing circular economy-driven material solutions.

## 2. Experimental Section

### 2.1. Materials

Recycled PET (RPET) was sourced from Alba Polyester Sdn. Bhd., Malaysia, and polyamide 11 (PA-11) was obtained from Arkema, based in King of Prussia, PA, USA. The epoxy functional-chain extender Joncryl^®^ ADR 4468 was supplied by BASF Corporation, Ludwigshafen, Germany. Halloysite nanotubes (HNTs), identified by product number 685445 and having the chemical composition Al_2_(OH)_4_Si_2_O_5_·nH_2_O, were acquired from Merck (Sigma-Aldrich) Sdn Bhd, SS7, 47301 Petaling Jaya, Selangor, Malaysia. Previous studies by Khan et al. [13,19] reported that RPET had a tensile strength of approximately 18.5 MPa and a flexural strength of 27.9 MPa, along with an Izod impact strength of 110 J/m. In contrast, PA-11 demonstrated superior mechanical performance, showing a tensile strength of 53 MPa and an impact resistance of 1371 J/m. According to the manufacturer’s data, Joncryl^®^ features a specific gravity of 1.08 at 25 °C, a molecular weight of 7250, and a glass transition temperature (Tg) of 59 °C. Its epoxy equivalent weight is 310 g/mol, and it contains more than 99% non-volatile material, as verified by gas chromatography. The technical specifications of the HNT filler used in this study are summarized in Table 1.

### 2.2. Extrusion Processing

The RPET/PA-11/Joncryl^®^/HNT nanocomposites were fabricated through a systematic melt blending process. A base blend of RPET and PA-11 was prepared in an 80:20 weight ratio, incorporating 2 phr of Joncryl^®^ 4468 as a compatibilizing agent. This concentration was previously optimized and validated in earlier work [19]. To examine the impact of HNT on composite performance, different loadings (0, 1, 2, 3, 4, and 5 phr) were introduced into the optimized polymer blend. Prior to compounding, all raw materials, including RPET (80 wt%), PA-11 (20 wt%), Joncryl^®^ (2 phr), and HNT (0–5 phr), were individually weighed using a precision digital balance. These components were then physically premixed to ensure initial homogeneity prior to extrusion. The premixed blend was slowly and carefully fed into the extruder, ensuring no material retention in the feed hopper and maintaining the target formulation throughout the process. Also, all components, RPET, PA-11, Joncryl^®^, and HNT were dried at 90 °C for 24 h in a Memmert ULM 500 oven (Gemini Lab, DG Apeldoorn, Netherlands) to remove moisture content. The melt blending was carried out using a co-rotating twin-screw extruder (Werner & Pfleiderer ZSK25, Stuttgart, Germany) with a screw diameter of 25 mm and a length-to-diameter (L/D) ratio of 40:1. The extruder featured six independently controlled heating zones and a vented barrel design. The screw configuration employed included a sequence of conveying elements, kneading blocks, and mixing elements strategically arranged to ensure effective melting, homogenization, and dispersion of the nanofillers within the polymer matrix. This setup provides both distributive and dispersive mixing capabilities, essential for achieving uniform nanocomposite materials. Processing was conducted at a screw speed of 80 rpm with a consistent feed rate of 4 kg/h to promote uniform HNT dispersion. The temperature across the zones ranged from 240 °C at the feed end to 290 °C at the die, ensuring optimal thermal conditions for the RPET/PA-11 system.

### 2.3. Injection Moulding and HNT Nanocomposite Formulations

Following the extrusion process, the nanocomposites were moulded into standard test specimens using an NI00B II injection moulding machine (JSW PTE LTD, Japan) in accordance with ASTM standards. The injection moulding was carried out with a temperature profile starting at 260 °C near the hopper and reaching up to 290 °C at the nozzle. Each moulding cycle lasted approximately 24 s. An injection pressure of 80 MPa was used, followed by a holding pressure of 60 MPa to ensure complete cavity filling and proper part formation. The mould temperature was maintained at about 60 °C to support optimal material flow, control the crystallization process, and enhance the dimensional stability of the moulded parts. Further insights into the properties of RPET, PA-11, and the compatibilizer Joncryl^®^, as well as the full methodology for sample preparation, are available in earlier publications [13,19]. The nanocomposite formulations used in this study are outlined in Table 2. Here, the term “phr” (parts per hundred resin) refers to the weight of filler added for every 100 g of polymer matrix. For instance, a 1 phr loading indicates that 1 g of filler was incorporated into 100 g of the polymer blend.

### 2.4. Characterization

Fourier-transform infrared (FTIR) spectroscopy was utilized to investigate the chemical interactions between the matrix and HNT nanofillers through PerkinElmer 1600 FTIR spectrophotometer, (available at AMTEC Laboratory UTM, Johor Bahru, Malaysia) operational with the attenuated total reflectance (ATR). The spectra were recorded over the wavenumber range of 4000 to 500 cm^−1^, with a resolution of 4 cm^−1^. Each spectrum was averaged over 32 scans to improve the signal-to-noise ratio. The morphological properties of composites were evaluated using a field emission scanning electron microscope (FESEM), Carl Zeiss Microscopy GmbH, Oberkochen, Germany. Prior to FESEM analysis, a thin platinum layer was deposited onto the sample surfaces using a sputter coater (Quorum Technologies Ltd., Laughton, East Sussex, UK) to enhance conductivity and imaging quality. The FESEM micrographs were taken at an acceleration voltage of 5 kV with an aperture size of 2 µm and magnifications of 20 K. The visualization of structural details on scales of 2 µm was obtained. The morphology of the blend (O phr HNT) was evaluated through scanning electron microscopy (SEM), (TM3000, Hitachi, Tokyo, Japan, available at UTM, Skudai Johor Bahru, Malaysia). The SEM imaging was performed at an accelerating voltage of 15 kV with a magnification of 1.0 K under high vacuum conditions, capturing micrographs at a scale of 100 µm. Tensile testing followed the ASTM D-638 standard [27] and was conducted using a Zwick/Roell Z020 universal testing machine. The test parameters included a pre-load of 0.01 MPa, a crosshead speed of 10 mm/min, and a secant modulus preset at 1%. Flexural strength measurements were carried out according to the ASTM D790 standard [28], using the same testing machine equipped with a 20 kN load cell. The flexural test employed a crosshead speed of 1 mm/min and a pre-load of 0.1 MPa. The test specimens for flexural analysis measured 127 mm in length, 12.3 mm in width, and 5 mm in thickness. The support span for the three-point bending test was set to 100 mm. The impact test was carried out using a Zwick Roell/HIT25P impact tester (available at N21 Laboratory UTM, Johor Bahru, Malaysia) in accordance with the ASTM D256-10 standard [29]. The pendulum energy used for the impact test was 11 joules. Each formulation was tested at least five times at room temperature. Prior to mechanical testing, the specimens were stabilized under controlled conditions maintained at 23 ± 2 °C and 50 ± 10% relative humidity for 48 h. This pre-conditioning step was essential to ensure moisture equilibrium, thereby minimizing the impact of ambient environmental factors on the accuracy and reliability of the mechanical test results. The thermal properties were analyzed using a PerkinElmer (STA 8000) (available at UNIPEM Laboratory UTM, Johor Bahru, Malaysia) differential scanning calorimeter (DSC) and thermogravimetric analysis (TGA). Differential scanning calorimetry (DSC) analysis was performed by heating the samples from room temperature up to 300 °C, followed by a controlled cooling process back to ambient temperature. All tests were conducted under a nitrogen atmosphere to minimize the risk of oxidation or thermal degradation during the thermal cycling. The nitrogen purge gas flow rate was set to 50 mL/min. No isothermal step was applied prior to the heating cycle. The temperature during the analysis was increased at a constant rate of 10 °C per minute. Thermogravimetric analysis (TGA) was carried out using a Perkin TGA 7 instrument (USA) (available at N18 Laboratory UTM, Johor Bahru, Malaysia) to assess the thermal stability and degradation characteristics of the nanocomposites. Approximately 5 mg of each sample was tested under a nitrogen flow of 50 mL/min to prevent oxidation throughout the experiment. The temperature was gradually increased from 30° C to 900 °C at a consistent rate of 10 °C per minute.

## 3. Results and Discussion

### 3.1. FTIR Analysis of Halloysite Nanocomposites

**Figure 1:**
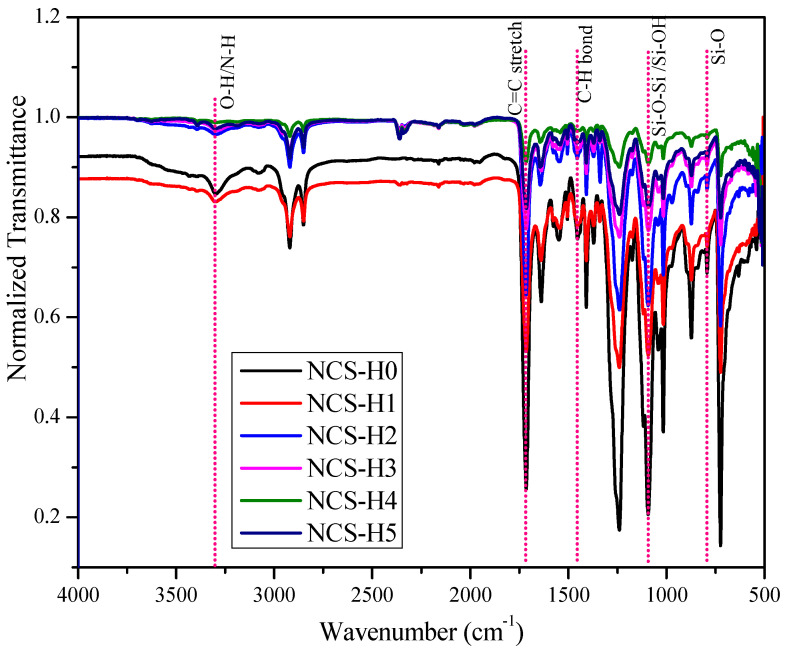
Normalized FTIR spectra of HNT nanocomposites.

**Figure 2:**
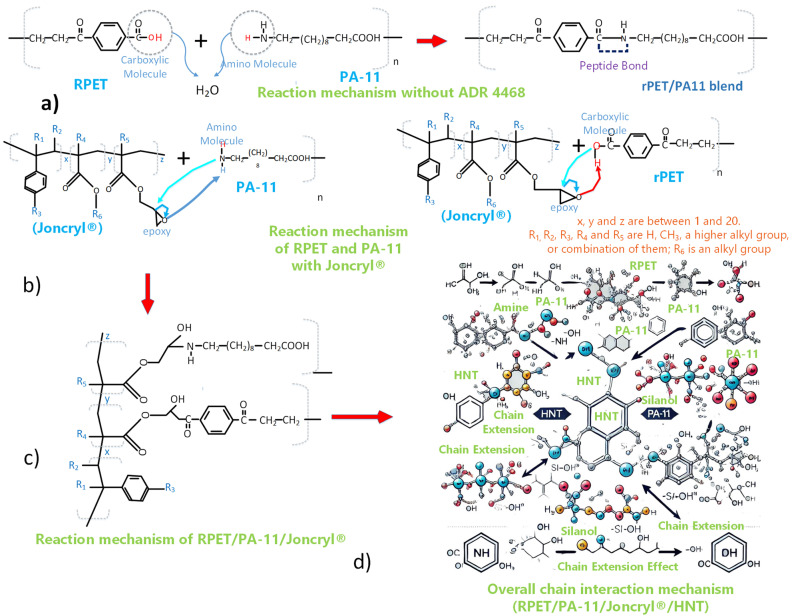
Reaction mechanism of (**a**) RPET/PA-11, (**b**) PA-11/Joncryl^®^ and RPET/Joncryl^®^, (**c**) RPET/PA-11/Joncryl^®^, (**d**) proposed chains’ interaction mechanism of RPET/PA-11/Joncryl^®^/HNT.

Figure 1 illustrates the FTIR spectra of the RPET/PA-11/Joncryl^®^ blend with varying HNT loadings (0–5 phr). All FTIR spectra are normalized to their respective maximum transmittance to ensure consistent comparison of spectral changes across formulations. The key characteristic bands are identified and annotated, including the broad O–H/N–H stretch (~3300 cm^−1^), ester C=O stretching (~1715 cm^−1^), and Si–O–Si or Si–OH vibrations (~1100 cm^−1^). The consistent presence and intensities of these bands indicate the successful integration of halloysite nanotubes without altering the primary chemical structure of the RPET/PA-11 matrix. Although the FTIR spectrum of the RPET/PA-11 blend without Joncryl^®^ is not included in this work, the comparative effect of Joncryl^®^ as a compatibilizer has been previously established and discussed in our earlier study [30]. Figure 2 shows the proposed chain interaction mechanism. The NCS-H0 formulation contains 0 phr HNT (control), while the NCS-H1 to NCS-H5 formulations have an HNT content of 1 to 5 phr. The spectra cover a wide range of wavenumbers (4000–500 cm^−1^), providing comprehensive data on the chemical bonding and interactions within the nanocomposites. The region (3700–3100 cm^−1^) corresponds to the O–H stretching vibrations of hydroxyl groups, mainly of HNT surfaces and possibly moisture adsorbed on HNT [31].

The peak around 3300 cm^−1^ corresponds to the N-H stretching vibrations of the PA-11 component. The intensity of this peak decreases with increasing HNT content, suggesting possible hydrogen bonding between the N-H groups of PA-11 and the hydroxyl groups of HNT [32]. The strong hydrogen bond interactions between the polymer and HNTs have also been described by other researchers [23]. In nanocomposites containing HNT, the observed spectral variations suggest meaningful interactions between the nanotubes and the surrounding polymer matrix. HNT appears to encourage molecular association, as evidenced by a reduction in peak intensity within the 3285 to 3400 cm^−1^ range. This decrease implies that hydroxyl groups may be involved in forming additional physical bonds. Such a trend, indicative of strengthened molecular interactions, is consistent with earlier studies [21]. The strong compatibility between HNT nanofillers and the compatibilized blend (NCS-H0) can likely be attributed to similar polarity characteristics, which enhance interfacial bonding. The strong absorption peak in the region (~1700–1750 cm^−1^) arises from the C=O stretching of the ester groups in RPET [33]. The position of this peak was relatively stable, but it was found that the intensity decreases slightly as HNT content increases, which indicates physical interactions between HNT and the RPET matrix restricting the vibrations of the C=O group. The FTIR wavelength between 1000 and 1100 cm^−1^ relates to the Si-O-Si and Si-OH vibrations of the HNT [34]. In the NCS-H4 and NCS-H5 spectra, these peaks are more noticeable than in the control sample (NCS-H0), which has weak or no peaks. Aliphatic hydrocarbons’ C-H bending vibrations and C-N stretching vibrations are represented by the peaks in this range (1300–1500 cm^−1^). [35]. The consistency in intensity suggests that the incorporation of HNT does not substantially alter the aliphatic framework of the polymer blend. The 500–900 cm^−1^ region is usually associated with other matrix-related vibrations and the bending vibrations of the Si-O groups from HNT. The presence of HNT and its interaction with the matrix is further demonstrated by the fact that the intensity of these peaks increases with increasing HNT content.

**Figure 3:**
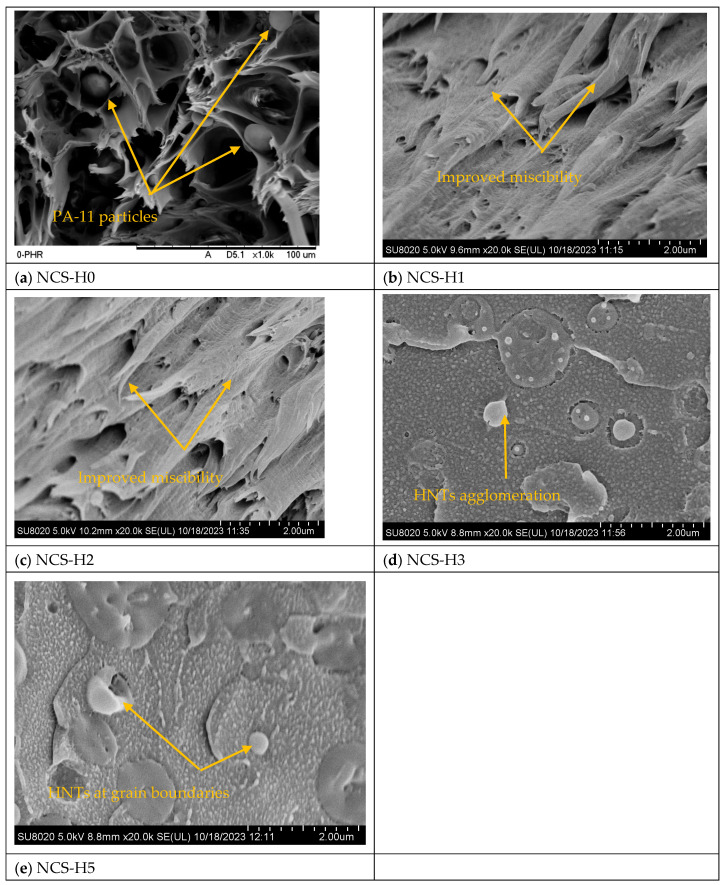
FESEM images, (**a**) NCS-H0, (**b**) NCS-H1, (**c**) NCS-H2, (**d**) NCS-H3, (**e**) NCS-H5, of nanocomposites.

The FTIR spectra disclose some variations in the strength of the C=O stretching (RPET) and N-H stretching (PA-11) peaks suggesting physical interactions and hydrogen bonds between the polymer matrix and the HNT surface (hydroxyl groups) [23]. A noticeable decrease in the FTIR absorption bands within the 3250–3390 cm^−1^ region points to enhanced hydrogen bonding, supporting earlier research that identified similar peak shifts as evidence of improved interactions between polymers and fillers [21,36]. The effective interaction among the components is further substantiated by the consistent and homogeneous distribution of HNTs, as shown in Figure 3b,c. Nevertheless, accurately identifying specific interactions within this spectral range remains complex due to overlapping absorption signals from both -NH and -OH functional groups. As shown in Figure 1, the FTIR spectra also reveal changes in the intensity of these -OH and -NH bands upon the addition of HNTs, indicating further alterations in the polymer–filler interface. This improved interaction is possibly due to the existence of the Joncryl^®^ compatibilizer improving interfacial adhesion between the RPET/PA-11 matrix and HNT. Joncryl^®^ improves miscibility between RPET and PA-11 by reacting with the hydroxyl (RPET) and amine (PA-11) groups to form a stable interface. The observed spectral changes are mainly due to physical interactions of HNT incorporation, with no evidence of chemical degradation or side reactions.

Figure 2d shows the proposed chains’ interaction mechanism with the incorporation of Joncryl^®^ and HNT improving structural integrity and interface interactions in the HNT-based nanocomposite. The reaction mechanism of RPET/PA-11, as shown in Figure 2a, was reported by researchers [13,21]. The reaction mechanisms of RPET with Joncryl^®^ and PA-11 with Joncryl^®^ were studied by Khan et al. and are shown in Figure 2b,c [19]. The presence of Joncryl^®^, a chain extender with multiple epoxy functional groups, facilitates the formation of amide and ester linkages by reacting with PA-11’s amine (-NH) groups and RPET’s carboxyl (-COOH) groups, respectively. These reactions contribute to improved polymer chain continuity and compatibility. Additionally, HNTs interact with the polymer matrix through hydrogen bonding (-OH) and silanol (-SiOH) interactions, reinforcing the network at the molecular level. In Figure 1, the FTIR spectra reveal distinct absorption bands near 3300 cm^−1^, corresponding to hydroxyl (-OH) stretching, along with Si-O-Si and Si-OH vibrations between 1000 and 1100 cm^−1^, confirming strong interactions between HNTs and the polymer backbone. Morphological analysis through FESEM (Figure 3c) displays a well-integrated nanostructure where HNTs are evenly dispersed, contributing to reduced phase separation and enhanced mechanical properties by reducing the PA-11 particle size evident in Figure 3a.

### 3.2. Morphology of HNT Nanocomposites

Figure 3 presents SEM and FESEM images of tensile fracture surfaces for RPET/PA-11/Joncryl^®^/HNT nanocomposites containing 0, 1, 2, 3, and 5 phr of HNT, captured at a magnification of 2 µm. The micrographs corresponding to formulations NCS-H0, NCS-H1, NCS-H2, NCS-H3, and NCS-H5 reveal the morphological characteristics and dispersion of halloysite nanotubes (HNTs) within the RPET/PA-11/Joncryl^®^ matrix. These images highlight the influence of HNT loading on the microstructure, providing insight into its role in enhancing reinforcement and overall composite integrity. The SEM image of NCS-H0 (Figure 3a) shows a smooth and continuous surface with some spherical PA-11 particles. The control sample lacks reinforcement and the presence of some voids between RPET and PA-11 contributes to its weak structural morphology. The FESEM image of NCS-H1 (Figure 3b) shows the initial incorporation of HNT particles into the RPET/PA-11 matrix. The surface morphology becomes rougher compared to NCS-H0, indicating the presence of HNT particles in the matrix. NCS-H1 demonstrates improved morphology and moderate reinforcement compared to the control sample. The coalescence of PA-11 particles in RPET/PA-11 blends without Joncryl^®^ was reported in our earlier study [13]. In the presence of Joncryl^®^, the number and size of dispersed PA-11 phases were significantly reduced due to improved compatibilization as reported [30]. The addition of 1 phr HNT further suppressed PA-11 coalescence by introducing a steric barrier effect, thereby improving phase dispersion and reducing porosity, as evident in the FESEM micrographs.

**Figure 4:**
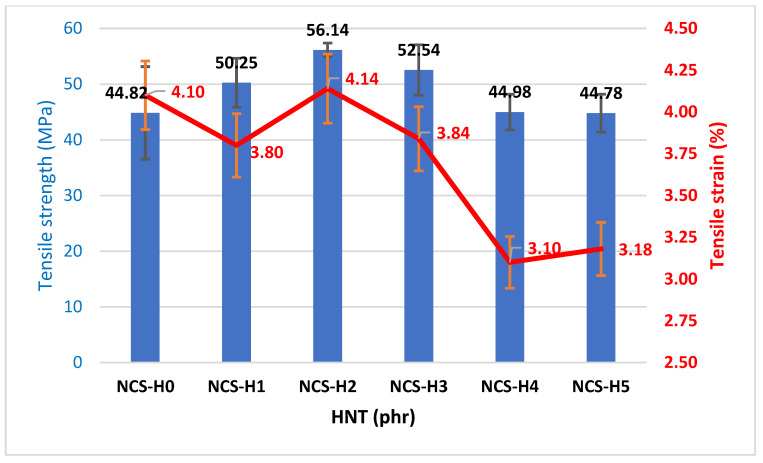
Tensile strength and strain (%) of HNT nanocomposites.

**Figure 5:**
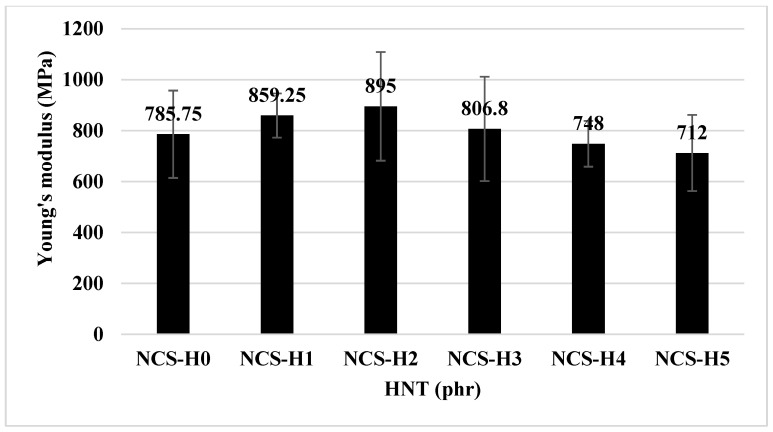
Young’s modulus of HNT nanocomposites.

**Figure 6:**
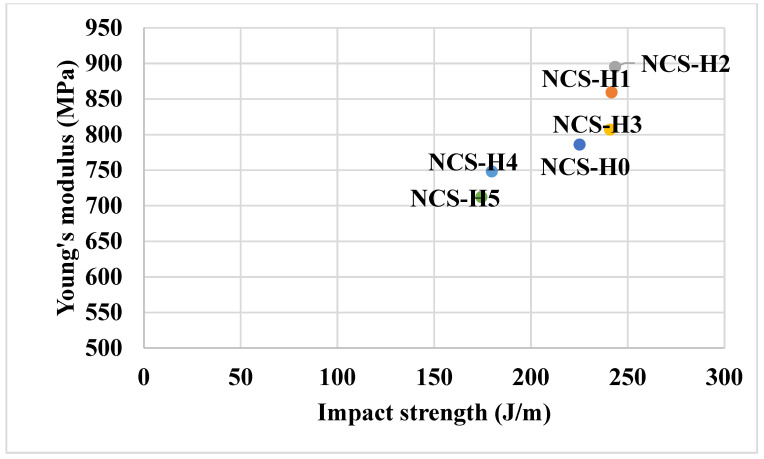
Young’s modulus vs. impact strength of HNT nanocomposites.

**Figure 7:**
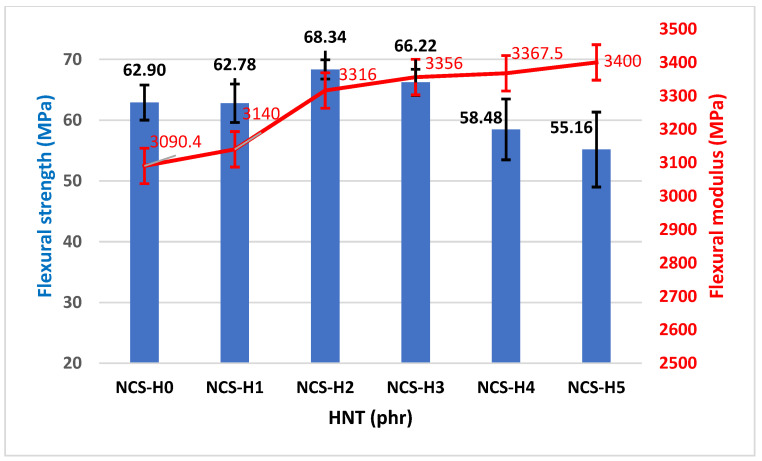
Flexural strength and flexural modulus of HNT nanocomposites.

The FESEM image of NCS-H2 (Figure 3c) reveals uniform dispersion of HNT particles throughout the polymer matrix. The HNT particles are well dispersed and embedded into the RPET/PA-11 matrix, indicating excellent interfacial interaction. The surface morphology is significantly rougher compared to NCS-H0 and NCS-H1, reflecting the strong adhesion between the HNT and the matrix. The absence of visible microvoids or phase boundaries suggests enhanced compatibility between RPET and PA-11 due to the synergistic effect of Joncryl^®^ compatibilizer and HNT reinforcement. The PA-11 particle that is distinct at NCS-H0 (Figure 3a) disappeared with the addition of HNT (Figure 3b), indicating improved compatibility. This is because of the existence of silanol groups on the surface of HNT fillers that take part in the physical interaction. The uniform distribution of HNT and strong interfacial interactions lead to enhanced structural integrity and reinforcement. This optimal dispersion maximizes the mechanical properties of the nanocomposite (Figure 4, Figure 5, Figure 6 and Figure 7). The FESEM image of NCS-H3 (Figure 3d) shows dispersion of HNT particles, with the beginning of localized clusters of HNT appearing. The surface roughness continues to increase, indicating enhanced interaction between the HNT and the matrix. However, agglomerates of HNT are observed, which may act as stress concentrators, limiting further improvements in morphology. The presence of smaller agglomeration suggests that the dispersion of HNT is slightly less uniform compared to NCS-H2. NCS-H5 (Figure 3e) shows significant HNT agglomeration at higher loading. At 5 phr HNT, excessive loading results in poor dispersion and agglomeration, affecting the morphology and structural integrity of the composite.

FESEM analysis shows that 2 phr HNT (NCS-H2) offers the best dispersion and morphology, resulting in uniform reinforcement and improved interfacial adhesion in the RPET/PA-11/Joncryl^®^ matrix. While NCS-H1 also has good morphology, NCS-H2 is characterized by the optimal composition to achieve improved structural integrity and mechanical reinforcement (Figure 4, Figure 5, Figure 6 and Figure 7). Excessive HNT loading (NCS-H3 to NCS-H5) leads to agglomeration, which reduces the effectiveness of the reinforcement. The morphological results of the nanocomposites are consistent with the HNT-based nanocomposites having different polymer matrices, as studied by researchers [37].

### 3.3. Tensile Testing of HNT Nanocomposites

Figure 4 depicts the tensile strength and strain (%) of RPET/PA-11/Joncryl^®^ nanocomposites containing varying halloysite nanotube (HNT) loadings, ranging from 0 to 5 phr. The control sample, i.e., NCS-H0 (0 phr), in Figure 4 shows the lowest tensile strength (44.825 MPa), reflecting less interfacial adhesion between the RPET and PA-11 phases due to lower compatibility as compared to HNT nanocomposites. The tensile strength increases with the incorporation of HNT. The addition of 1 phr of HNT significantly improves the tensile strength to 50.25 MPa, indicating initial strengthening and better stress transfer due to the presence of HNT. The maximum tensile strength (56.14 MPa) is observed at 2 phr HNT. This is due to the uniform distribution and strong interfacial bonding between HNT and the matrix, which enable efficient load transfer. At higher HNT loadings from NCS-H3 to NCS-H5 (3–5 phr), the tensile strength decreases (44.98–44.78 MPa), which is likely due to agglomeration, affecting the effective interaction between HNT and the matrix and introduces stress concentrators. The enhanced tensile strength observed for NCS-H2 is further corroborated by the FESEM morphological analysis shown in Figure 3c. This explains its superior tensile strength. NCS-H5 exhibits significant agglomeration as observed in the morphology (Figure 3e), resulting in poor stress transfer and reduced tensile strength. The increased tensile strength values in 2 phr HNT nanocomposite were more significant than the close and related work of researchers working on RPET/HDPE/alumina nanocomposites [38]. They observed an increase in the tensile strength of RPET with the addition of HDPE, alumina nanoparticles, and compatibilizers, and its maximum value was 33.5 ± 2.3 MPa. Similar results were reported by Khan et al. (2024) for the tensile strength of RPET/PA-11/Joncryl^®^/sepiolite at 41.3 MPa [20]. Similarly, the tensile strength of RPET/PA-11/GNP recently reported by Unsia et al. (2025) [21] was 37.85 ± 2.13 MPa, while this work achieved a higher tensile strength of 56.14 MPa with HNT nanofillers.

The control sample of tensile strain (%) shown in Figure 4 shows the tensile strain (4.1%), which indicates higher flexibility due to the absence of HNT particles. The tensile strain (%) decreases slightly with the addition of 1 phr of HNT; however, it was found to be highest at 2 phr of HNT (NCS-H2) due to improved miscibility. However, beyond 2 phr of HNT, the tensile strain (%) reduced due to possible agglomeration of HNT filler at the higher content. The higher tensile strain (%) of NCS-H2 nanocomposites reflects a balance between rigidity and flexibility. The tensile strain of NCS-H4 and NCS-H5 reduces significantly with the increase in HNT content (3.1–3.18%), indicating that excessive HNT content decreases the matrix’s flexibility due to particle agglomeration and increased stiffness. The moderate strain (%) at NCS-H2 aligns with its optimal HNT dispersion (Figure 3c), which avoids excessive rigidity while maintaining reinforcement. The reduced strain (%) at higher loadings (NCS-H4 and NCS-H5) is consistent with the observed agglomeration (Figure 3d,e), which increases stiffness and reduces elongation. A similar decreasing trend in tensile strain was reported by other researchers with the addition of sepiolite, the tensile strain (%) was reported at 2.76 with the addition of 2 phr sepiolite while at 8 phr it was recorded at 1.96 [20]. Similarly the tensile strain (%) was reported as 2.14 ± 0.09 with the addition of 2 phr GNP [21].

Figure 5 and Figure 6 show Young’s modulus and Young’s modulus vs. impact strength of all RPET/PA-11/Joncryl^®^/HNT nanocomposites. Young’s modulus of RPET/PA-11, with 2 phr Joncryl^®^ and 0 phr HNT (NCS-H0), was recorded at 785.75 MPa. The control sample (NCS-H0) has the lowest modulus indicating weak interfacial adhesion and poor load distribution. By adding 1 to 2 phr of HNT nanofillers (NCS-H1 to NCS-H2), the tensile modulus showed an increasing trend, with the highest value recorded at NCS-H2 (895 MPa), which may be attributed to enhanced stiffness and potential load transfer due to the presence of dispersed HNT. However, with a further increase in the HNT content from 3 to 5 phr (NCS-H3 to NCS-H5), the modulus decreases. NCS-H5 shows the lowest value (712 MPa) among the reinforced samples, probably due to agglomeration reducing the effective reinforcement. The uniform distribution of HNT in NCS-H2 increases the stiffness, which is consistent with the morphology results (Figure 3c). The agglomeration in NCS-H5 disrupts the matrix continuity (Figure 3e), leading to reduced modulus values.

Figure 6 representing Young’s modulus vs. impact strength of HNT nanocomposites reveals that NCS-H2 achieves the highest combination of modulus and impact strength, highlighting its superior mechanical performance. NCS-H5 shows the lowest impact strength and modulus, confirming the detrimental effect of HNT at high loadings. These results demonstrate the importance of achieving a uniform distribution of HNT in nanocomposites to maximize reinforcement and mechanical performance. NCS-H2 (2 phr HNT) proves to be an optimal formulation that balances stiffness and toughness. These are crucial properties that determine the application of nanocomposites. These results of the HNT nanocomposites are consistent with those reported by other researchers examined for sepiolite-based nanocomposites [20,30].

### 3.4. Flexural Properties of HNT Nanocomposites

**Figure 8:**
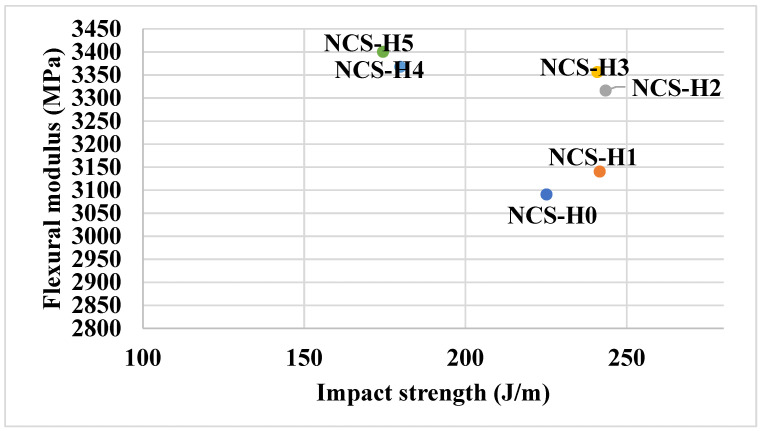
Flexural modulus vs. impact strength of HNT nanocomposites.

Figure 7 and Figure 8 represent the flexural strength, flexural modulus, and flexural modulus versus impact strength for the different ratios of HNT nanocomposites. The control sample (NCS-H0) has a flexural strength of 62.9 MPa, indicating the baseline performance without HNT reinforcement (0 phr). A slight decrease to 62.78 MPa is observed for nanocomposites with 1 phr HNT (NCS-H1), which is attributed to limited reinforcement due to partial HNT dispersion. NCS-H2 with 2 phr HNT showed the maximum flexural strength of 68.34 MPa, which is attributed to uniform HNT dispersion and strong interfacial interaction. The flexural strength of HNT nanocomposites having HNT from 3 to 5 phr (NCS-H3 to NCS-H5) slightly decreases to 66.22 MPa (NCS-H3) and further drops to 55.16 MPa (NCS-H5), likely due to agglomeration of HNT particles, which disrupts load transfer and reduces strength. The optimal dispersion observed in NCS-H2 from FESEM analysis (Figure 3c) supports its superior flexural strength. The accumulation of HNT at grain boundaries NCS-H5, as observed in morphology (Figure 3e), explains the reduced strength at higher HNT content. The observed trend in flexural strength is similar to the trend in tensile strength (Figure 4). The flexural strength of HNT nanocomposites is higher as compared to the flexural strength of RPET/PA-11 reinforced with graphene and sepiolite fillers [21].

As depicted in Figure 7, the flexural modulus demonstrates an upward trend with the progressive addition of HNT. The unfilled blend (NCS-H0) exhibits a flexural modulus of 3090.4 MPa, reflecting the baseline rigidity of the RPET/PA-11 matrix. Upon the incorporation of HNT, the modulus increases to 3316 MPa in NCS-H2, suggesting enhanced stiffness attributed to uniform HNT dispersion and stronger filler–matrix interactions. This increasing trend continues, with NCS-H5 reaching a modulus of 3400 MPa, even though a decline in tensile strength and elongation is observed at higher filler content. This suggests that even poorly dispersed HNT contributes to stiffness but at the expense of other properties. The consistent increase in modulus with HNT loading correlates with the presence of rigid HNT particles that resist deformation. However, the agglomeration in NCS-H5 (Figure 3e) affects other mechanical properties. NCS-H2 and NCS-H3 have the best combination of flexural modulus and impact strength (Figure 8), reflecting the balance between stiffness and toughness. NCS-H4 and NCS-H5 formulations exhibit high modulus but reduced impact strength, consistent with other results. The uniform dispersion in NCS-H2 (Figure 3c) supports its superior mechanical performance across all parameters. The clustering observed in NCS-H5 (Figure 3e) explains the reduced impact strength despite a high modulus. Overall, the flexural properties of RPET/PA-11/Joncryl^®^ nanocomposites improve with the addition of HNT up to an optimal concentration (2 phr). These results highlight the importance of uniform dispersion to maximize the reinforcing potential of the HNT nanocomposite and are consistent with other researchers’ results for optimal HNT dispersion at lower levels [39].

### 3.5. Impact Strength of HNT Nanocomposites

**Figure 9:**
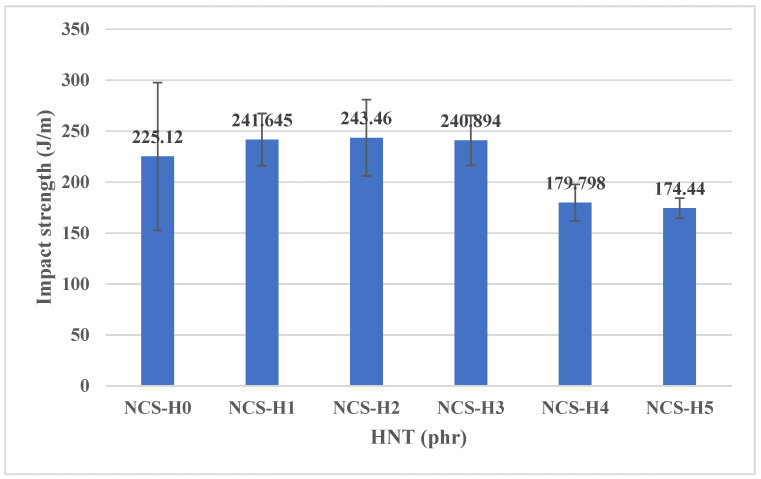
Impact strength of HNT nanocomposites.

Figure 9 depicts the impact strength of RPET/PA-11/Joncryl^®^ nanocomposites with varying HNT concentrations ranging from 0 phr (NCS-H0) to 5 phr (NCS-H5). The results showed that the incorporation of HNT initially increased the impact strength, with the highest value observed at 2 phr HNT (NCS-H2). However, the impact strength decreased at higher HNT concentrations (4–5 phr).

The control sample (NCS-H0) showed an impact strength of 225.12 J/m, reflecting the inherent toughness of the RPET/PA-11 matrix without reinforcement. With the addition of 1 phr HNT (NCS-H1), the impact strength increased to 241.64 J/m, indicating improved energy absorption due to the incorporation of HNT, which acted as a reinforcing agent and distributed the impact energy more effectively. At 2 phr HNT (NCS-H2), the impact strength peaked at 243.46 J/m, which corresponded to the optimal HNT dispersion observed in the morphology results (Figure 3c). The uniform distribution of HNT within the matrix enabled efficient stress transfer and energy dissipation, resulting in improved impact resistance. At higher HNT concentrations, specifically 3 phr (NCS-H3), the impact strength showed a slight decrease to 240.89 J/m, which could be attributed to the onset of HNT agglomeration. Agglomerates serve as points of stress concentration, which diminishes the composite’s capacity to effectively absorb and disperse impact energy. The reduction became more evident at 4 phr (NCS-H4) and 5 phr (NCS-H5), where the impact strength decreased to 179.79 J/m and 174.44 J/m, respectively. The significant decrease was directly related to the accumulation of HNT at grain boundaries as observed in the FESEM images (Figure 3d,e). These accumulations disrupted the continuity of the matrix and hindered stress transfer, resulting in brittle behaviour under impact loads.

The impact strength results closely matched the observed trends in tensile and flexural properties. NCS-H2, which exhibited superior tensile strength, flexural strength, and optimal elongation values, also exhibited the best impact resistance due to the uniform distribution of HNT and strong interfacial bonding. The agglomeration at higher HNT loadings (NCS-H4 and NCS-H5) not only reduced the tensile and flexural strength but also affected the toughness of the nanocomposites. A similar trend of impact strength with HNT nanofiller in another polymer matrix was observed by other researchers [37]. The impact strength of HNT nanocomposites is much higher than the impact strength of GNPs (195.725 ± 11.63) having 2 phr of GNPs in the same blend [21].

### 3.6. Differential Scanning Calorimetry (DSC)

**Figure 10:**
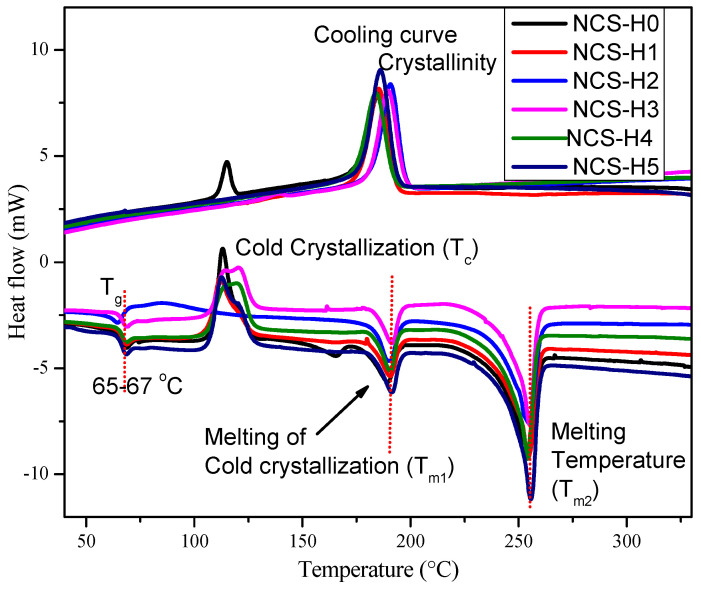
DSC curves of HNT nanocomposites.

Figure 10 and Table 3 present the DSC analysis results. The glass transition temperature (Tg) of the RPET/PA-11 nanocomposites was found to lie in the range of 63.93 to 66.79 °C, positioned between the Tg of neat RPET (approximately 70–80 °C) [40] and that of pure PA-11 (around 50–55 °C). The observed intermediate Tg suggests potential interfacial interactions between the RPET and PA-11 phases, likely enhanced by the incorporation of Joncryl^®^ as a compatibilizer and the presence of HNT nanofillers. While RPET and PA-11 are typically immiscible, the addition of compatibilizers and nanoparticles can improve interfacial adhesion, limit polymer chain mobility, and consequently influence the Tg. Comparable trends have been reported in earlier studies involving phase-separated polymer blends, where interfacial enhancements contributed to modified thermal transition behaviour [20]. The T_g_, observed as a step change in the heat flow curve at approximately 63.93–66.79 °C, remained relatively stable across all formulations. The stable T_g_ with the addition of HNT indicated improved interfacial interactions between the matrix and the nanotubes, which limited the molecular mobility. The nanocomposites with 2 phr HNT (NCS-H2) showed slightly lower T_g_ at 63.93 °C due to the uniform distribution of HNT, which increased the toughness of the matrix.

In the exothermic phase of the DSC analysis, as the temperature increases from 30 °C to 350 °C, certain polymers like recycled PET (RPET) within the nanocomposites exhibit a noticeable cold-crystallization peak (Tc) around 113–120 °C (Figure 10 and Table 3). This upward transition suggests that the presence of HNTs promotes nucleation and accelerates crystallization processes. A rightward (higher temperature) shift in Tc suggests that crystallization becomes more difficult or delayed, often due to restricted chain mobility, strong filler–matrix interactions, or altered nucleation behaviour. Interestingly, when 2 phr of HNTs is introduced (NCS-H2), the observed reduction in cold-crystallization temperature from 113.19 °C in NCS-H0 to 86.27 °C in NCS-H2 (Table 3) indicates enhanced nucleation facilitated by the HNTs, which promote crystallization at lower temperatures. This behaviour suggests that HNTs provide effective heterogeneous nucleation sites and alter polymer chain mobility. The shifting of the cold-crystallization peak at 2 phr HNT to lower temperature suggests that this filler concentration optimally facilitates crystallization during heating and is a sign of excellent filler dispersion and effective nucleation behaviour. As the HNT content increases to 3–5 phr, Tc shifts to higher temperatures; this trend suggests that, at higher filler loadings, the restricted polymer chain mobility and potential agglomeration of HNTs hinder crystallization, thereby requiring higher thermal energy for cold crystallization to occur. After the crystallization temperature (Tc), a cold-crystallization melting peak (Tm1) is observed as a downward slope on the thermogram, as shown in Figure 10. This thermal transition observed near 190 °C (Tm₁) is considered to result from the melting of cold-crystallized RPET, a phenomenon widely reported in the literature [30]. Given that the melting point of PA-11 also typically falls within the 185–190 °C range, it is possible that this peak represents an overlap between the melting of PA-11 and that of cold-crystallized RPET phase formed during the heating process. A comparable cold-crystallization behaviour has been previously reported in studies involving RPET/PA-11/GNP nanocomposites, highlighting similar effects of nanoparticle inclusion on crystallization dynamics [21]. The T_m1_, represented by the endothermic peaks near 190 °C, remained consistent across all samples. The stability of T_m1_ indicated that the overall crystal structure of the RPET/PA-11 blend was preserved regardless of the HNT content. Continued heating reveals the final melting point of the nanocomposite (T_m2_).

Crystallinity (Xc) plays a crucial role in determining the mechanical performance of polymer composites. The degree of crystallinity was calculated based on the RPET content (80 wt%) in the blend, consistent with previous studies on RPET/PA-11-based nanocomposites. Given that Joncryl^®^ and HNT were added in small quantities (2 phr and 0–5 phr, respectively), their effect on the overall mass was considered negligible for the purpose of this calculation. Additionally, PA-11 was not analyzed separately for crystallinity, as it primarily contributed to the amorphous phase under the studied processing conditions. This methodology was adopted to ensure meaningful comparison with previously reported RPET/PA-11 systems reinforced with GNP, sepiolite, and hybrid nanofillers [16,21]. The crystallinity (Xc) for each nanocomposite is calculated based on the crystallization peaks obtained from the DSC cooling curves, applying the following formula:

(1) $$Xc\left(\%\right)=\frac{\Delta H}{\begin{array}{c} \Delta Ho\times 0.8 \end{array}}\times 100$$

In the applied equation, ΔH denotes the normalized melting enthalpy derived from the crystallization peak in the DSC cooling curve. ΔHo refers to the heat of fusion for 100% crystalline PET, commonly cited as 140 J/g in published studies [16]. The multiplier 0.8 reflects the 80 wt% content of RPET within the nanocomposite formulation. Therefore, any changes observed in crystallinity are mainly influenced by the RPET phase and its interaction with the incorporated HNT. The Xc of NCS-H0 (4.19%) increased to 29.47% (NCS-H1), 29.31% (NCS-H2), 28.57% (NCS-H3), 31.83% (NCS-H4), and 30.88% (NCS-H5). The crystallinity percentage (Xc) shows a notable rise with the incorporation of HNT. The control sample, NCS-H0, exhibits the lowest crystallinity at 4.19%, highlighting the limited crystalline structure of the unfilled RPET/PA-11 blend. The marked increase in Xc upon the addition of HNT reflects its strong nucleating effect within the RPET/PA-11/Joncryl^®^ matrix. These results suggest that HNT effectively promotes crystallization by serving as a nucleating agent. The same is supported by the higher stiffness of the nanocomposites as the tensile and flexural modulus (Figure 5 and Figure 7) increased, reflecting the stiffness.

Overall, the DSC results demonstrated that the addition of HNT influenced the thermal transitions of RPET/PA-11/Joncryl^®^ nanocomposites. These observations were consistent with the morphological results, which showed uniform HNT dispersion in NCS-H2 (Figure 3c) and agglomeration at higher loadings (Figure 3d,e). The Xc values indicate that HNTs function as a nucleating agent, improving crystallization. The shifting of Tc towards higher temperature with HNT content signifies a crystallization-promoting effect also proven from the crystallinity calculation of the cooling curves. The DSC results are consistent with the results of Wieczorek et al. (2024), who investigated HNT filler incorporation in high-density polyethylene (HDPE) [41]. A minor crystallization peak was detected during the cooling cycle for NCS-H0, likely corresponding to the PA-11 phase. However, this peak was not observed in the nanocomposite formulations (NCS-H1 to NCS-H5), which may be attributed to the restricted coalescence and enhanced dispersion of PA-11 due to the presence of HNT, as supported by the SEM observations (Figure 3).

### 3.7. Thermogravimetric Analysis (TGA)

**Figure 11:**
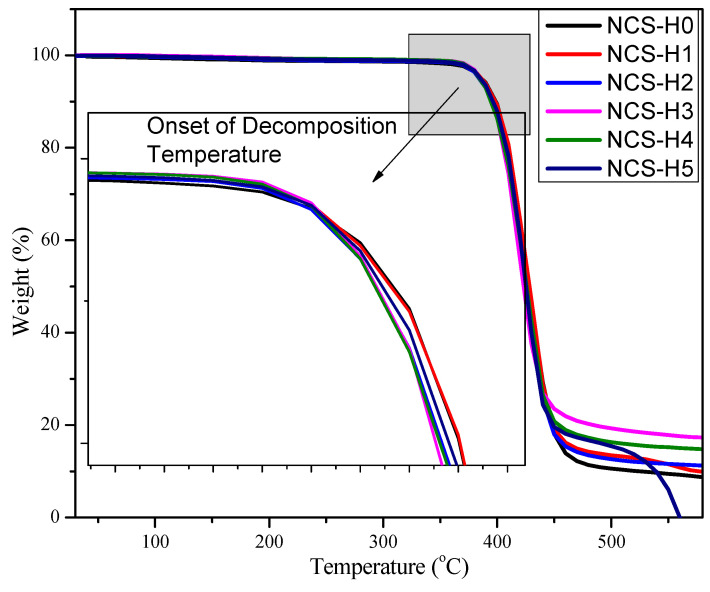
TGA curves of HNT nanocomposites.

Figure 11 illustrates the thermal stability of RPET/PA-11/Joncryl^®^ nanocomposites containing 0 to 5 phr of HNT while Table 4 shows the temperature at 10% and 50% weight loss. The weight-loss profiles were analyzed to determine the effect of HNT on the thermal decomposition behaviour of the nanocomposites, with key stages of degradation examined and integrated with the morphology, mechanical properties, and crystallinity results. The TGA curves showed minimal or no weight loss below 100 °C, corresponding to the evaporation of residual moisture. All formulations exhibited a similar behaviour in this region, reflecting the absence of significant thermal events in this temperature range. The consistent trend suggested that the addition of HNT did not introduce excessive moisture into the matrix, which was supported by the uniform dispersion observed at lower HNT loadings (NCS-H1 and NCS-H2) in Figure 3b,c.

The greatest thermal degradation of the HNT nanocomposites occurred between 350 °C and 450 °C, primarily due to the decomposition of the matrix components (RPET and PA-11). The control sample (NCS-H0) exhibited the lowest thermal stability, with the onset of degradation observed at approximately 360 °C. Incorporating HNT into the nanocomposite improved the onset temperature, with NCS-H2 demonstrating the highest thermal stability at 370 °C. This enhancement was attributed to the barrier effect of well-dispersed HNT, which restricted the mobility of polymer chains and delayed the release of volatile degradation products. Similar findings in a polypropylene matrix were reported by Wieczorek et al. (2024), showing that HNT incorporation reduced weight loss at higher temperatures [41]. The improvement in thermal stability is also supported by the crystallinity data obtained from DSC analysis (Figure 10). The control sample exhibited a low crystallinity of 4%, while the addition of HNT significantly enhanced crystallinity in the nanocomposites (28.57% to 31.83%). HNT acted as a nucleating agent, promoting crystallization within the polymer matrix, leading to a more ordered and thermally stable structure. This increased crystallinity contributed to the higher onset degradation temperature observed in TGA results, particularly for NCS-H2. However, at higher HNT loadings (NCS-H4 and NCS-H5), a slight decrease in thermal stability was observed, indicated by a reduction in the onset temperature. This behaviour was consistent with the reduced interactions observed in the morphology (Figure 3d,e), which reduced the effectiveness of HNT in the matrix. The reduced interaction not only impacted the barrier effect but also influenced the crystallinity of the nanocomposites. The DSC results showed that excessive HNT loading led to a slight decrease in crystallinity, aligning with the reduced thermal stability observed in TGA.

The residual weight at 600 °C reflected the char formation and thermal stability of the nanocomposites. The control sample (NCS-H0) had the lowest residue, indicating poor thermal stability. With increasing HNT content, the residual weight increased proportionally, with NCS-H5 having the highest char residue. The increase in residue with HNT content highlighted the inert nature of HNT, which contributed to thermal resistance and char stability. The improved thermal stability at 2 phr HNT (NCS-H2) was consistent with the uniform dispersion observed in the FESEM image (Figure 3c). The homogeneously distributed HNT acted as an effective thermal barrier, reducing heat transfer and delaying decomposition [24]. The enhanced crystallization temperature observed in DSC for NCS-H2 (Figure 10) suggested improved nucleation by uniformly dispersed HNT. This increase in crystallinity contributed to the improved thermal stability of the nanocomposite at 2 phr. The trends in thermal stability were consistent with other results, where NCS-H2 exhibited superior performance due to optimal HNT dispersion and strong interfacial bonding. Researchers reported similar results of improved thermal stability with HNT incorporation in a different polymer matrix (polypropylene), which showed less weight loss at higher temperatures [41]. The homogeneously distributed HNTs acted as effective thermal barriers, reducing heat transfer and delaying decomposition. Other researchers also reported that 2.0 phr HNT significantly improved the thermal stability of PLA/CSR/HNT due to a higher shift in the degradation temperature [42]. The enhanced crystallization temperature observed in DSC for NCS-H2 suggested improved nucleation by uniformly dispersed HNT. This increase in crystallinity contributed to the enhanced thermal stability of the nanocomposite at 2 phr. The marginal decrease in thermal stability at higher loadings (NCS-H4 and NCS-H5) was consistent with the agglomeration observed in these formulations, which created weak points and reduced the barrier effect. At higher HNT loadings (NCS-H4 and NCS-H5), the thermal stability decreased slightly, which was reflected in a slight reduction in the onset temperature. This behaviour was consistent with the reduced tearing observed in the morphology (Figure 3d,e), which reduced the effective interaction between the HNT and the matrix.

Overall, the TGA results showed that the incorporation of HNT improved the thermal stability of RPET/PA-11/Joncryl^®^ nanocomposites. This improvement was attributed to the barrier effect of well-dispersed HNT and its contribution to char formation.

## 4. Conclusions

This study demonstrated the successful development of RPET/PA-11/Joncryl^®^ nanocomposites reinforced with halloysite nanotubes (HNTs), achieving significant improvements in mechanical, thermal, and morphological properties. The findings highlight that an optimal HNT loading of 2 phr (NCS-H2) yields the most balanced enhancement across all performance metrics. The mechanical characterization revealed a remarkable increase in tensile strength, peaking at 56.14 MPa for NCS-H2, a 26% improvement over the control sample. Similarly, flexural strength reached its highest value of 68.34 MPa at 2 phr HNT, marking a substantial reinforcement effect. Young’s modulus also exhibited a significant increase, attaining 895 MPa for NCS-H2, confirming the enhanced stiffness due to uniform HNT dispersion. Impact strength followed a similar trend, with NCS-H2 achieving the highest value of 243.46 J/m, reinforcing the role of well-dispersed HNT in improving energy absorption and toughness. However, at higher loadings (4–5 phr), mechanical performance declined due to HNT agglomeration, leading to stress concentrators and phase discontinuities.

Thermal analysis through DSC and TGA further validated the improvements. The glass transition temperature (T_g_) exhibited a slight increase, confirming restricted polymer chain mobility due to efficient matrix–filler interactions. The thermal stability was significantly enhanced, with NCS-H2 showing the highest onset degradation temperature of 370 °C, compared to 360 °C for the control sample. The increased residue content at 600 °C for NCS-H2 further confirmed its superior thermal resistance. Morphological evaluation through FESEM provided visual confirmation of the well-dispersed HNTs at 2 phr, demonstrating excellent interfacial adhesion with the polymer matrix. NCS-H2 exhibited a homogeneous microstructure with minimal voids and uniform filler dispersion, directly correlating with its superior mechanical and thermal properties. In contrast, higher HNT loadings led to particle aggregation, which reduced reinforcement efficiency and structural integrity. The NCS-H2 has excellent mechanical and thermal properties, and therefore it can play a vital role in advanced applications in the area of engineering plastics and sustainable materials. These results provide a solid basis for future research to optimize filler distribution strategies to further improve the performance of recycled RPET/PA-11 nanocomposites.

Future studies might include DSC analysis based on the second heating cycle to eliminate the effects of thermal history, allowing for a more precise evaluation of glass transition temperature, cold-crystallization behaviour, and crystallinity changes in the RPET/PA-11/HNT nanocomposites. Furthermore, future studies will incorporate EDS mapping to confirm and visualize the spatial distribution of HNT within the RPET/PA-11 matrix, thereby providing further insights into filler dispersion and interfacial interaction.

## 5. The Significance of the Study

This research is representing a breakthrough in the development of high-performance, sustainable recycled PET based polymer nanocomposites. A novel approach to integrate HNT into the RPET/PA-11 matrix addressed critical challenges in plastics recycling, structural strengthening, and thermal stability. This study provides an innovative solution by using the synergistic effects of Joncryl^®^ as a compatibilizer and HNT as a reinforcing agent with enhanced mechanical properties, excellent heat resistance and optimized interfacial adhesion. The research is pioneering the use of RPET blended with bio-based PA-11, ensuring an environmentally friendly approach to advanced polymer engineering in a sustainable way. The significant improvement recorded in the properties of tensile strength, impact strength, and heat resistance properties with optimized HNT loadings underlines the effectiveness of this nanocomposite, which exceeds that of the conventional polymer blends. The results of this study open up new paths for sustainable plastics development with potential applications in automotive, aerospace, packaging, and high-performance structural components. The ability to tailor the properties of nanocomposites through controlled filler dispersion and chain extension mechanisms shows that this approach is feasible for industrial applications on a large scale. As industry increasingly prioritizes environmentally friendly materials, this research sets standards for the next generation of high-performance plastic nanocomposites that offer both scientific advancements and industrial relevance.

## Figures and Tables

**Table 1:** Properties of HNT.

Properties	Nominal Value Unit
Molecular weight	294.19 g/mol
Colour	white
pH	4.5–7.0
Length	1–3 microns
Diameter	30–70 nanometres
Pore volume	1.26–1.34 mL/gm

**Table 2:** Formulations of HNT nanocomposites.

Formulations	RPET (wt%)	PA-11 (wt%)	Joncryl^®^ (phr)	HNT (phr)
NCS-H0	80	20	2	0
NCS-H1	80	20	2	1
NCS-H2	80	20	2	2
NCS-H3	80	20	2	3
NCS-H4	80	20	2	4
NCS-H5	80	20	2	5

**Table 3:** DSC results of HNT nanocomposites.

Formulations	T_g_ (°C)	T_c_ (°C)	T_m1_ (°C)	T_m2_ (°C)	Xc (%)
NCS-H0	66.72	113.19	189.24	253.53	4.19
NCS-H1	66.79	111.97	190.42	253.84	29.47
NCS-H2	63.93	86.27	189.78	254.26	29.31
NCS-H3	66.94	120.32	191.16	254.75	28.57
NCS-H4	66.79	119.30	190.43	253.67	31.83
NCS-H5	66.76	112.64	191.05	255.07	30.88

**Table 4:** Indicating temperature at 10% and 50% mass loss.

Formulation	10% Loss Temp (°C)	50% Loss Temp (°C)
NCS-H0	~395	428.26
NCS-H1	~395	428.26
NCS-H2	~395	426.60
NCS-H3	~395	425.10
NCS-H4	~395	426.60
NCS-H5	~395	436.55

## Data Availability

Currently, the raw data that are critical to reproducing these results are not available for public sharing, as they are an essential part of an ongoing doctoral project. The raw data will be confidential until the project is completed and published.
